# Predicting anticancer synergistic drug combinations based on multi-task learning

**DOI:** 10.1186/s12859-023-05524-5

**Published:** 2023-11-27

**Authors:** Danyi Chen, Xiaowen Wang, Hongming Zhu, Yizhi Jiang, Yulong Li, Qi Liu, Qin Liu

**Affiliations:** 1 School of Software Engineering, Tongji University, Shanghai, 201804 China; 2 Translational Medical Center for Stem Cell Therapy and Institute for Regenerative Medicine, Shanghai East Hospital, Bioinformatics Department, School of Life Sciences and Technology, Tongji University, Shanghai, 200092 China

**Keywords:** Multi-task learning, Deep neural networks, Autoencoder, Anticancer treatment, Synergistic drug combinations

## Abstract

**Background:**

The discovery of anticancer drug combinations is a crucial work of anticancer treatment. In recent years, pre-screening drug combinations with synergistic effects in a large-scale search space adopting computational methods, especially deep learning methods, is increasingly popular with researchers. Although achievements have been made to predict anticancer synergistic drug combinations based on deep learning, the application of multi-task learning in this field is relatively rare. The successful practice of multi-task learning in various fields shows that it can effectively learn multiple tasks jointly and improve the performance of all the tasks.

**Methods:**

In this paper, we propose MTLSynergy which is based on multi-task learning and deep neural networks to predict synergistic anticancer drug combinations. It simultaneously learns two crucial prediction tasks in anticancer treatment, which are synergy prediction of drug combinations and sensitivity prediction of monotherapy. And MTLSynergy integrates the classification and regression of prediction tasks into the same model. Moreover, autoencoders are employed to reduce the dimensions of input features.

**Results:**

Compared with the previous methods listed in this paper, MTLSynergy achieves the lowest mean square error of 216.47 and the highest Pearson correlation coefficient of 0.76 on the drug synergy prediction task. On the corresponding classification task, the area under the receiver operator characteristics curve and the area under the precision–recall curve are 0.90 and 0.62, respectively, which are equivalent to the comparison methods. Through the ablation study, we verify that multi-task learning and autoencoder both have a positive effect on prediction performance. In addition, the prediction results of MTLSynergy in many cases are also consistent with previous studies.

**Conclusion:**

Our study suggests that multi-task learning is significantly beneficial for both drug synergy prediction and monotherapy sensitivity prediction when combining these two tasks into one model. The ability of MTLSynergy to discover new anticancer synergistic drug combinations noteworthily outperforms other state-of-the-art methods. MTLSynergy promises to be a powerful tool to pre-screen anticancer synergistic drug combinations.

## Background

Anticancer treatment with personalization is a challenge in modern medicine [1]. It is particularly important to match the right therapy to specific cancer while considering the patient’s characteristics for the improvement of treatment efficacy [2]. In personalized medicine, drug combination therapy has the significant advantages of less toxicity, better efficacy, and lower possibility of drug resistance than monotherapy [3]. Drug interactions include synergy, antagonism, and additive effect, which means the effect of a drug combination is greater than, less than, and equal to the sum of each drug, respectively [4, 5]. Therefore, a drug combination is not necessarily more beneficial, and it is a meaningful task to find drug combinations with synergistic effects.

Clinical trials are the primary way in which most synergistic drug combinations are discovered. However, it is cost-intensive and requires large amounts of time and labor, and may bring unnecessary or even harmful treatment to patients [6, 7]. In contrast, high-throughput screening (HTS) techniques do not require patient trials, but instead, use experimentally grown cancer cell lines to screen for drugs that may be used in therapy. Although HTS is now an effective approach to discovering drug combinations, it is not feasible to completely test the huge drug combination space through HTS experiments due to objective reasons such as economic cost and experimental difficulty [8]. The wide application and rapid development of computer technology provide researchers with another thinking.

Faced with the huge screening space, many computational methods for screening drug combinations have emerged in recent years. They are mainly divided into two categories: hypothesis-driven and data-driven [5]. The former includes systems biology methods [9], biomolecular network-based methods [10], etc. However, the datasets used in hypothesis-driven computational methods are small and these methods are restricted to certain pathways, targets, or cell lines [11]. Moreover, assumptions used to guide model construction can not be guaranteed correct, and the reliability is insufficient [3].

Data-driven models are developed based on features of known synergistic drug combinations [5], among which machine learning (ML) models including Random Forest [12], Gradient Tree Boosting [13], Extremely randomized tree [14], and Tensor factorization [15] are competitive. With the continuous enrichment of experimental data in the field of anticancer drug research and the rapidly growing demand for learning large-scale datasets and high-dimensional features like gene expression data, a special class of ML methods, deep learning (DL), is increasingly favored by researchers. DL methods can automatically learn suitable feature representations from data without manually constructing features, which has advantages in processing large-scale synergy datasets [3]. DeepSynergy [11], AuDNNsynergy [16], PRODeepSyn [17] and TranSynergy [18] employ deep neural networks (DNNs) for the synergy prediction of drug combinations. Furthermore, PRODeepSyn utilizes graph convolutional neural networks (GCNs) to extract the topological structure from omics data and protein–protein interaction network data. Recently, some researchers have introduced multi-task learning [5, 19] in synergistic drug combinations prediction by exploring correlation and valuable information of multiple tasks.

Multi-task learning (MTL) is a subfield of machine learning in which multiple learning tasks are solved at the same time. It aims to leverage useful information from multiple related tasks to improve the performance and generalization ability of all the tasks [20]. MTL is widely used in natural language processing, speech recognition, and computer vision [21], but its application in predicting anticancer synergistic drug combinations has just started. Chen et al. [5] proposed a drug synergy prediction model DSML, which combined drug target prediction and drug synergy prediction into a unified framework, and enriched model training information by reconstructing drug–target protein interactions, to accurately and effectively predict drug synergy. However, it is difficult to obtain excellent computational performance when reconstructing drug–target interactions, since the set of protein–protein interactions is sparse and noisy. Kim et al. [19] developed a model using transfer learning technology in anticancer drug synergy prediction. The model was pre-trained on data-rich tissues using multi-task learning, aiming to learn information from these tissues. And then they applied knowledge transfer to predict drug synergy in understudied, experimentally-data-deficient, but critical tissues. Nevertheless, They only regard monotherapy sensitivity prediction as an auxiliary task of drug synergy prediction in the model, thus focusing on synergy results but the sensitivity results.

In this paper, we design an anticancer synergistic drug combinations prediction model MTLSynergy based on multi-task learning and deep neural networks. MTLSynergy tackles two crucial prediction tasks in cancer treatment: the synergy prediction of drug combinations and the sensitivity prediction of monotherapy. Both tasks are jointly addressed and optimized in the same model, with a focus on delivering high-quality results for both drug synergy and monotherapy sensitivity predictions. We utilize drug molecular fingerprints and descriptors as drug features, and RNA-Seq TPM gene expression data as cell line features, which are extremely accessible and do not require excessively complex processing. We leverage drug features and cell line features to pre-train a drug encoder and a cell line encoder separately, thereby reducing the dimension of features and obtaining encoded representations that are easier and more efficient to learn. Subsequently, these encoded representations serve as inputs to the DNNs of MTLSynergy enabling the prediction of drug combination synergy scores and monotherapy sensitivity scores. Additionally, MTLSynergy combines two classification tasks instead of training classification models separately, thus mitigating computational overhead and time cost. MTLSynergy adopts the O’Neil dataset [22] for training and testing. Through a series of experiments, we demonstrate that MTLSynergy outperforms other state-of-the-art (SOTA) methods. We further conduct ablation experiments and hyperparameters sensitivity analysis to elucidate the impact of the multi-task learning and autoencoder on the performance of drug synergy prediction and monotherapy sensitivity prediction. In addition, we apply MTLSynergy to predict previous study cases, finding that prediction results are consistent with these studies. Overall, MTLSynergy is proven to be a powerful method for pre-screening anticancer synergistic drug combinations.

## Materials and methods

### Datasets

We utilize the large-scale synergy dataset published by O’Neil et al. [22] in 2016 for training and evaluating our model. The dataset covers the results of experimental testing of 583 different drug combinations against 39 human cancer cell lines. In experiments, each sample was assayed four times adopting a $4\times 4$ dosing regimen, and the cell growth rate relative to the control group after 48 h was measured. Preuer et al. [11] calculated Loewe Additivity [23] values of 23,062 samples in O’Neil. We take the average of replicates, resulting in 22,737 data samples. In order to avoid data leaks, all samples are equally divided into five folds according to drug combinations, which means a certain drug combination only exists in the specified fold. Since drug combinations that show high synergy are more attractive in clinical anticancer treatment, they receive more attention in research and are more worthy of being tested in practice. Therefore, in the classification task of drug synergy prediction, we regard samples with synergy scores higher than 30 as strong synergistic combinations (positive class) while classifying the remaining as the negative class, referring to Preuer et al. [11].

Sensitivity scores of 38 different drugs on 39 cancer cell lines, 1482 items in total, are derived from DrugComb [24, 25]. DrugComb is a comprehensive portal that aggregates publicly available anticancer drug combination synergy data, integrating dozens of drug synergy experimental researches including O’Neil. Besides, it also provides sensitivity data of each drug in a drug combination on specific cell line. In DrugComb, the sensitivity of a single drug is characterized as a dose–response curve with IC50 and RI (Relative Inhibition) values [25]. For our purposes, we utilize RI values provided by DrugComb as monotherapy sensitivity scores. RI is the normalized area under the dose–response curve transformed by log10, which is more robust than other modalities in characterizing drug sensitivity [26]. While IC50 and EC50 are usually relative indicators that often depend on the concentration range tested, RI shows the overall inhibitory effect of the drug relative to the control group, facilitating comparisons of drug response in different concentration ranges [25]. In the classification task of monotherapy sensitivity prediction, we adhere to the practice of Kim et al. [19] by establishing a threshold of 50 to demarcate positive and negative samples.

We perform drug filtration on the DrugComb dataset, eliminating corrupted or illogical data that could adversely affect our experiments. This process ensures that every drug possesses a valid SMILES expression and is amenable to the extraction of drug fingerprints. Additionally, we systematically eliminate drugs lacking synergy data within the DrugComb dataset. These drugs lack corresponding combinations and samples, potentially impeding the prediction of synergistic drug combinations. We finally obtain 3118 drugs (including 38 O’Neil drugs) and 175 cell lines (including 39 O’Neil cell lines) from DrugComb to pre-train a drug autoencoder and a cell line autoencoder. Comprehensive information regarding the autoencoders is provided in section “Autoencoder”. Further details of drugs and cell lines are shown in Additional file 1 and Additional file 2, respectively.

### Drug features

**Fig. 1:**
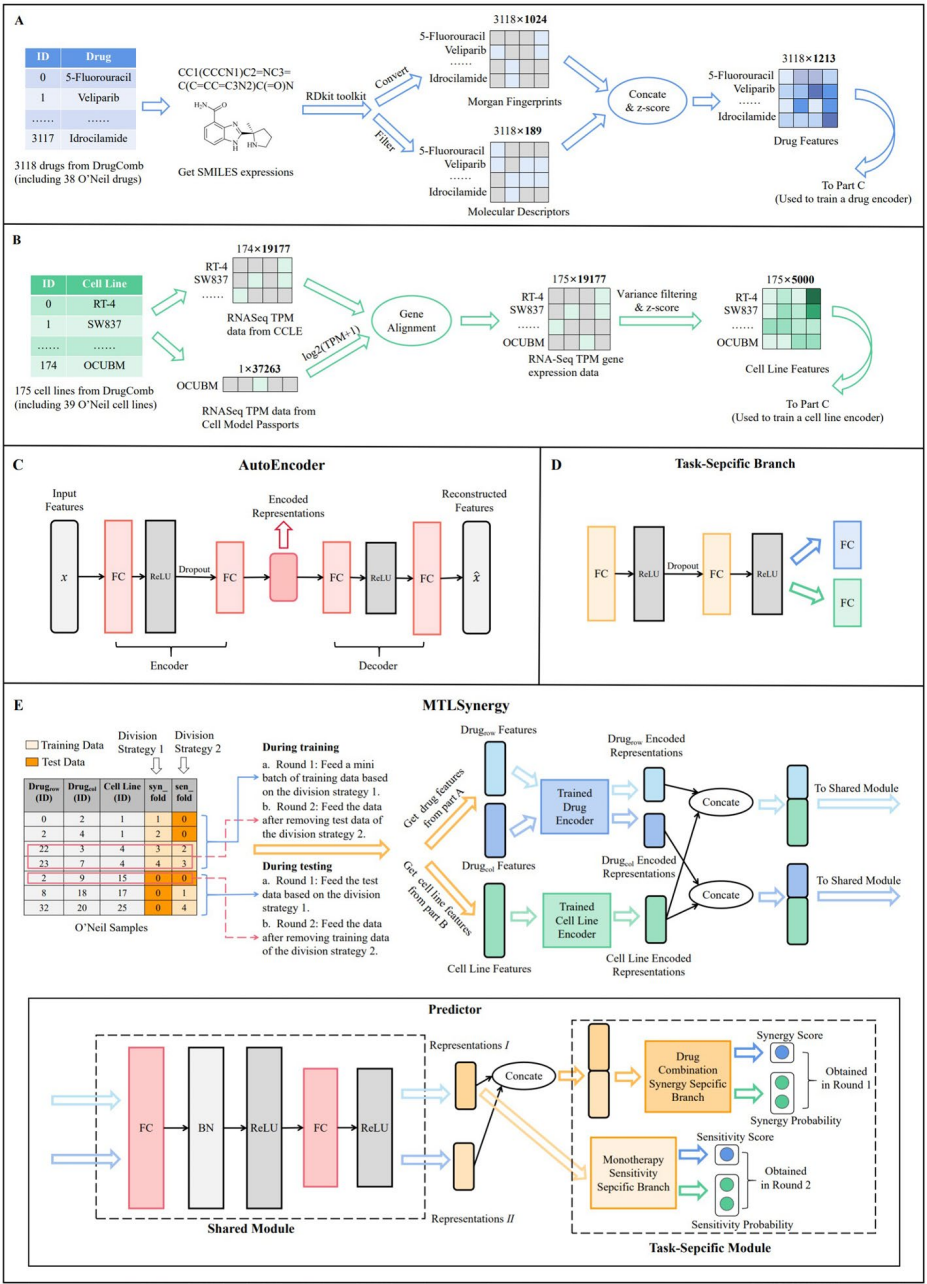
Overview of MTLSynergy. **A** Preprocessing of drug features. **B** Preprocessing of cell line features. **C** Structure of autoencoder. **D** Structure of task-specific branch. **E** Structure of MTLSynergy

Morgan fingerprints [27] and molecular descriptors [28] are adopted to capture the structure and physicochemical properties of drugs, which are calculated by RDkit toolkit [29] based on SMILES expressions. We generate Morgan fingerprints with a radius of 3 and convert them into a 1024-dimensional binary vector. The molecular descriptors of 3118 drugs with empty values or zero variance are filtered out, resulting in a 189-dimensional vector for each drug. Afterward, we concatenate two types of aforementioned vectors and then carry out z-score normalization to generate a 1213-dimensional vector for each drug as drug features. The process is shown in Fig. 1A.

### Cell line features

We acquire RNA-Seq TPM gene expression data of 19,177 genes for 174 cell lines from CCLE [30], which include 38 O’Neil cell lines but lack the cell line OCUB-M. Therefore, we obtain RNA-Seq TPM data for OCUB-M from another database, Cell Model Passports [31], and perform log2(TPM+1) transformation consistent with CCLE. Since 113 genes from CCLE can not align with Cell Model Passports, averages of 174 cell lines for 113 genes are utilized to fill in the missing gene data of OCUB-M. For a total of 175 cell lines, we screen out 5000 genes with the largest variance and perform z-score normalization to construct 5000-dimensional vectors as cell line features. The process is shown in Fig. 1B.

### MTLSynergy

We propose a multi-task learning-based method MTLSynergy which jointly learns two crucial prediction tasks of cancer treatment in the same framework. MTLSynergy handles the synergy prediction of drug combinations and the sensitivity prediction of monotherapy, including both regression and classification tasks. The model is structured into two parts: the autoencoder part (Fig. 1C) and the predictor part (Fig. 1E). Autoencoders are initially pre-trained adopting the aforementioned drug features and cell line features, respectively. The trained encoders are then utilized to transform drug and cell line features in a low-dimensional dense space. Subsequently, the predictor is trained by leveraging these encoded representations to predict synergy scores, sensitivity scores and their respective classification results. Further details of MTLSynergy are introduced in the following sections.

#### Autoencoder

We build autoencoders to obtain encoded representations with dimensionality reduction, which are beneficial to discover notable information from drug or cell line features and improve learning efficiency as well as reduce computational overhead. The autoencoder consists of multi-layer feed-forward neural networks, as shown in Fig. 1C. The first two fully connected (FC) layers are an encoder with dropout, and the last two are a decoder. ReLU activation function is used between FC layers to introduce nonlinear properties. The objective function involves minimizing the error between the original input features *x* of the encoder and the reconstructed output features *x̂* of the decoder, and the formula is defined as follows:

1 $$\begin{aligned} Loss_{AE}(x,{\hat{x}})=\frac{\sum _{i=1}^n{(x_{i}-\hat{x_{i}})^2}}{n} \end{aligned}$$

where *n* is the number of training samples.

We utilize 1213-dimensional drug features to pre-train a drug autoencoder and leverage 5000-dimensional cell line features to pre-train a cell line autoencoder. The drug encoded representations from the trained drug encoder and the cell line encoded representations from the trained cell line encoder are subsequently adopted as inputs to the predictor.

#### Predictor

The predictor part of the model shown in Fig. 1E consists of two main modules: the shared module and the task-specific module. The shared module has two FC layers equipped with batch normalization and a ReLU activation function. It accepts the concatenation of encoded representations of one drug and one cell line as input and generates a representation of shared knowledge applicable to both drug synergy prediction and monotherapy sensitivity prediction tasks. In cases where a sample contains two drugs and a cell line, the shared module will produce two distinct representations labeled as *I* and *II* for each drug–cell line pair.

Branch structures are present within the task-specific module following the shared module, as shown in Fig. 1D. MTLSynergy is trained on two tasks: drug synergy prediction and monotherapy sensitivity prediction. For the drug synergy prediction task, the representations *I* and *II* are initially concatenated and subsequently fed into the drug combination synergy specific branch, which encompasses multiple FC layers utilizing ReLU activation function and dropout. This branch includes two output layers: one responsible for generating the synergy score of a drug combination, and the other for producing the probability distribution pertaining to the synergy classification, utilizing the softmax activation function.

For the monotherapy sensitivity prediction task, only the representation *I* is utilized as input for the monotherapy sensitivity specific branch. This branch shares a structural resemblance with the drug combination synergy specific branch. The final outputs consist of the sensitivity score of a single drug and the probability distribution for the sensitivity classification.

Since drug combinations presented in order $drug_{row}-drug_{col}$ or $drug_{col}-drug_{row}$ should not be differentiated, each sample is included in training in both presentation orders. The predicted synergy score for a drug combination on a given cell line is computed as the average of the predicted results obtained in both orders. This training approach ensures that drugs at the $drug_{row}$ position in drug combinations remain consistent with drugs at the $drug_{col}$ position. Consequently, only the sensitivity scores of the $drug_{row}$ on a cell line need to be predicted in the monotherapy sensitivity prediction task.

We adopt mean square error (MSE) and binary cross entropy (BCE) as loss functions for two regression tasks and two classification tasks respectively. Let the total number of training samples be represented as *n*. The BCE function, applied to the real label *y* and the predicted probability distribution *ŷ,* is defined as follows:

2 $$\begin{aligned} Loss_{bce}(y,{\hat{y}})=-\frac{\sum _{i=1}^{n}y_{i}log\hat{y_i}+(1-y_i)log(1-\hat{y_i})}{n} \end{aligned}$$

The total loss of the prediction model is computed as the sum of the four loss functions, as follows:

3 $$\begin{aligned} Loss_{tol}=Loss_{mse}^{syn}+Loss_{mse}^{sen}+Loss_{bce}^{syn}+Loss_{bce}^{sen} \end{aligned}$$

the marks *syn* and *sen* represent the drug synergy prediction task and the monotherapy sensitivity prediction task, respectively.

## Experimental setup

### Method comparison

We conduct a comparative analysis between MTLSynergy and other SOTA methods, for the prediction of synergistic drug combinations. Our evaluation is based on the large-scale synergy dataset published by O’Neil et al. [22], adopting a rigorous 5-fold cross-validation approach. The O’Neil samples are partitioned into five distinct folds, each characterized by unique drug combinations. Consequently, drug combinations present in one fold do not overlap with those in the other four folds. Furthermore, all methods employ identical data splits for the delineation of training, validation, and test sets. MTLSynergy is compared with four DL models including DeepSynergy [11], PRODeepSyn [17], TranSynergy [18], and AuDnnSynergy [16], as well as two widely adopted ML models including Random Forest [12] and Gradient Tree Boosting [13]. The evaluation outcomes for the four DL models are sourced directly from their original research papers. Additionally, we have made available source code links for these methods in our repository. It should be noted that TranSynergy excludes some cell lines, resulting in its evaluation being conducted on the incomplete O’Neil dataset. For two ML models, we employ the Random Forest method and the Gradient Tree Boosting method encapsulated in the Sklearn 1.0.2 python package. These models are trained independently, utilizing feature data that aligns with MTLSynergy. Detailed hyperparameter settings for the two ML methods can be found in Additional file 3. We present only evaluation results of synergy prediction in section “Method comparison”, as all compared methods are single-task in nature. Evaluation results for sensitivity prediction are showcased in sections “Ablation study” and “Hyperparameters sensitivity analysis”. In section “Ablation study”, we introduce ablation experiments conducted on several MTLSynergy variants. These experiments are designed to demonstrate the effectiveness of multi-task learning and autoencoders in the context of two prediction tasks. In section “Hyperparameters sensitivity analysis”, we elucidate how alterations in the dimensionality of encoders impact the performance of MTLSynergy.

### Performance metrics

For regression tasks, we adopt mean squared error (MSE) as the main evaluation metric, and we also compute the root mean squared error (RMSE) and Pearson correlation coefficient (PCC) between the predicted scores and the actual values. For classification tasks, we employ evaluation metrics including the area under the receiver operating characteristic curve (ROC-AUC), the area under the precision–recall curve (PR-AUC), and accuracy (ACC).

### Model settings

For autoencoders, we establish the output dimension of the drug encoder as $c_{drug} = 128$ and the cell line encoder as $c_{cell} = 256$. During predictor training, we mainly adjust hyperparameters including hidden layer size and learning rate. The optimal combination is determined by grid search. The available choices for the size of the initial FC layer in the shared module consist of {2048, 4096, 8192}, while the options for the learning rate are {0.0005, 0.0001, 0.00005}. To mitigate overfitting during training, we implement an early stopping mechanism. Training will be halted if, within 100 iterations, the total loss on the validation set ceases to decrease or if the number of iterations exceeds 500. In such cases, we preserve the model with the lowest loss.

For the drug synergy prediction task, we partition 22,737 samples into five folds based on distinct drug combinations, following the approach outlined by Preuer et al. [11]. While this strategy guarantees that each drug combination resides in only one designated fold, it results in each fold encompassing all drugs in the dataset. Consequently, we once more categorize the samples into five folds, this time based on different drugs, for the monotherapy sensitivity prediction task. This strategy aligns with the example samples depicted in Fig. 1E. In the training phase, we feed the data into the model in mini-batches, adhering to the first division strategy, and compute the losses pertaining to the drug synergy prediction task. To prevent data leakage in the monotherapy sensitivity prediction task, we subsequently exclude the test data associated with this task from the mini-batch. We do this based on the marks of the second division strategy, and we feed this filtered data back into the predictor and calculate the losses. In the final step, we compute the total loss and execute a gradient update. This process remains consistent during testing. Initially, we make predictions for the synergy results on the test set, and subsequently, we compute sensitivity results. Importantly, we remove the training data associated with this task from the test set prior to sensitivity calculation to prevent data contamination.

**Table 1:** Results of the Method Comparison on the Regression Task

Method	MSE	RMSE	PCC
MTLSynergy	**216.47**±**37.32**	**14.66**±**1.26**	**0.76**±**0.02**
PRODeepSyn	229.49±42.81	15.09±1.37	0.75±0.02
TranSynergy*	231 ± 21	–	0.75±0.02
AuDnnSynergy	241.12±43.52	15.46±1.44	0.74±0.03
DeepSynergy	255.49	15.91±1.56	0.73±0.04
Gradient Tree Boosting	274.88±44.97	16.53±1.33	0.69±0.02
Random Forest	360.15±51.32	18.93±1.34	0.57±0.02

Boldface indicates the best value of each column in a particular metric

*TranSynergy excludes certain cell lines from O’Neil dataset

**Table 2:** Results of the Method Comparison on the Classification Task

Method	ROC-AUC	PR-AUC	ACC
MTLSynergy	0.90±0.02	0.62±0.05	**0.94±0.01**
PRODeepSyn	0.90±0.03	0.63±0.05	0.93±0.01
TranSynergy*	**0.91±0.01**	**0.63±0.01**	–
AuDnnSynergy	0.91±0.02	0.63±0.06	0.93±0.01
DeepSynergy	0.90±0.03	0.59±0.06	0.92±0.03
Gradient Tree Boosting	0.90±0.01	0.56±0.05	0.93±0.01
Random Forest	0.87±0.02	0.52±0.05	0.91±0.01

Boldface indicates the best value of each column in a particular metric

*TranSynergy excludes certain cell lines from O’Neil dataset

**Fig. 2:**
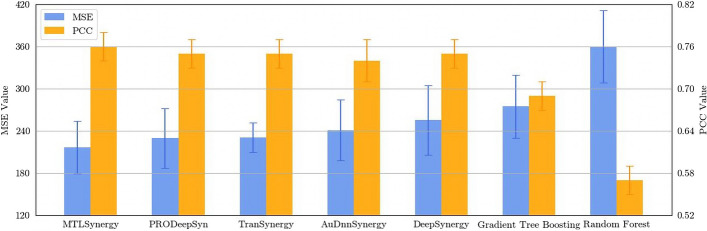
Comparison of different methods on MSE and PCC

## Results

### Method comparison

**Fig. 3:**
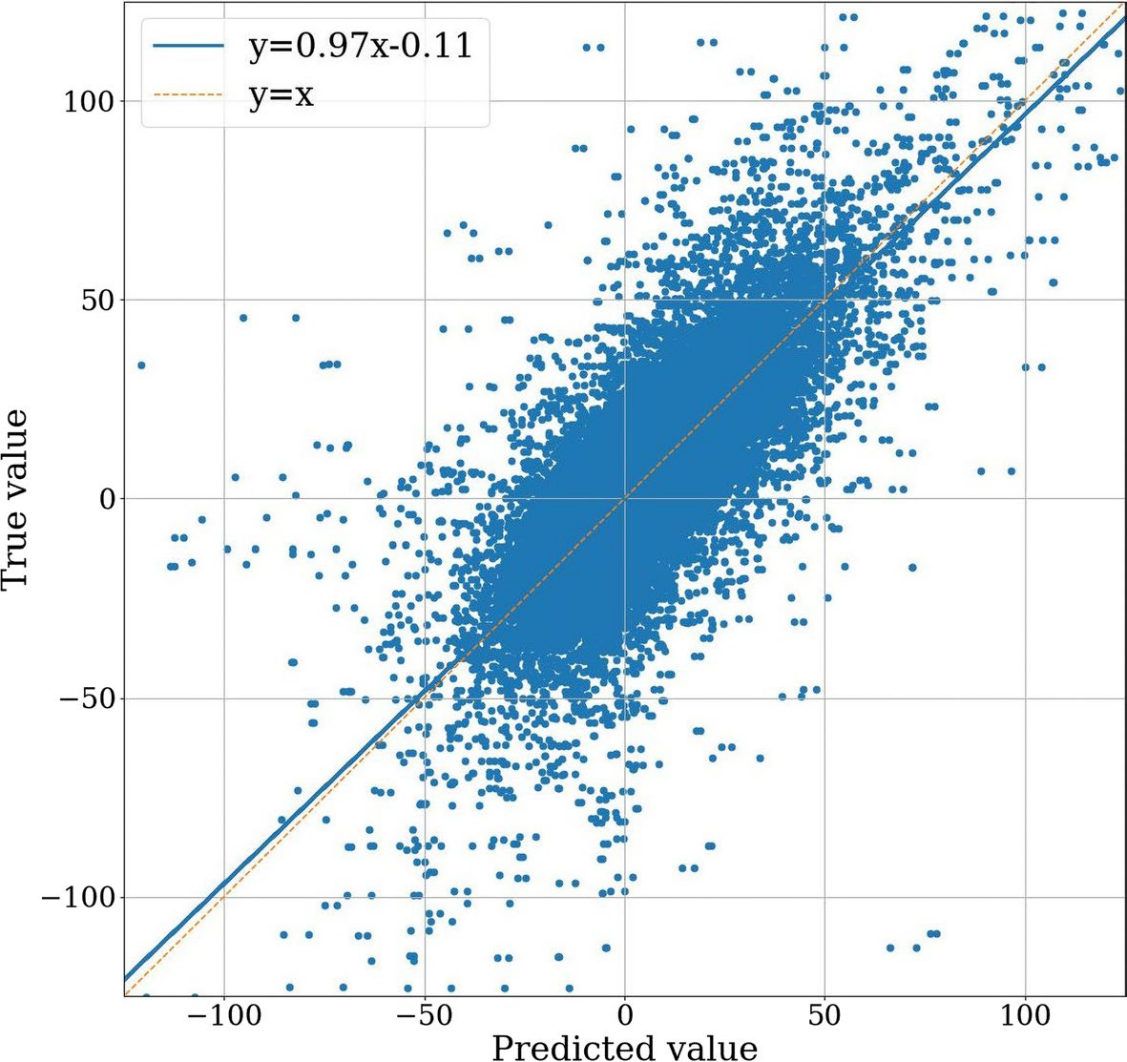
Scatter plot of the predicted synergy scores and the ground truth

Table 1 presents the regression results from the comparative analysis conducted for the drug synergy prediction task. Among the enumerated drug synergy prediction models, MTLSynergy stands out with the lowest MSE value, measuring 216.47. This marks a 5.67% reduction compared to PRODeepSyn, a 15.27% reduction compared to DeepSynergy, and a notable 21.25% reduction compared to Gradient Tree Boosting. Additionally, MTLSynergy achieves the highest PCC value of 0.76 among the compared methods. Figure 2 provides a visual representation that clearly illustrates the substantial advantages of MTLSynergy in terms of both MSE and PCC. Figure 3 reveals the correlation between the predicted synergy scores generated by MTLSynergy and the ground truth values. We conduct a linear regression analysis utilizing the least squares method, resulting in a straight line characterized by a slope of 0.97 and an intercept of $-$0.11. This line closely aligns with the reference line $y=x$, demonstrating that the predicted values of MTLSynergy on the drug synergy prediction task exhibit a robust linear correlation with the actual values. These results prove that MTLSynergy significantly outperforms other SOTA methods.

We also report the classification results in Table 2, considering that many studies approach the drug synergy prediction task from a classification perspective. We derive the evaluation results of MTLSynergy based on the synergy probability it generates. We find that MTLSynergy exhibits proximity to the best results in terms of ROC AUC and PR AUC and achieves the highest ACC of 0.94. The distinctions among the top four methods on the classification task is inconspicuous. One potential explanation for this observation is that treating the task of identifying synergistic drug combinations as a binary classification problem is an oversimplification that may make the results less realistic [32]. Overall, MTLSynergy performs remarkably on the regression task and shows competitiveness on the classification task.

Additionally, we conduct evaluations of MTLSynergy, DeepSynergy, Gradient Tree Boosting, and Random Forest using identical feature data in the *Leave Drugs Out* scenario (samples are split to make that drugs present in the test set are absent from the training set) and in the *Leave Cell Lines Out* scenario (samples are split to make that cell lines seen in the test set are not included in the training set), respectively. In the *Leave Drugs Out* scenario, MSEs for all methods fall within the range of 430 to 461, while in the *Leave Cell Lines Out* scenario, MSEs span from 379 to 510. The detailed results are presented in Additional file 3. The predictive performance of all methods in these two scenarios is massively worse compared to their performance in the *Leave Drug Combinations Out* scenario (our main experiments). This phenomenon can be attributed to the limited amount of training examples in terms of only 38 distinct drug types and 39 cell line types involved. The evaluation results of the two machine learning methods are better than those of the two deep learning methods. This discrepancy can be attributed to the capacity of traditional machine learning methods to learn effectively from limited available data, whereas deep learning algorithms typically demand a more extensive dataset for proficient training.

**Fig. 4:**
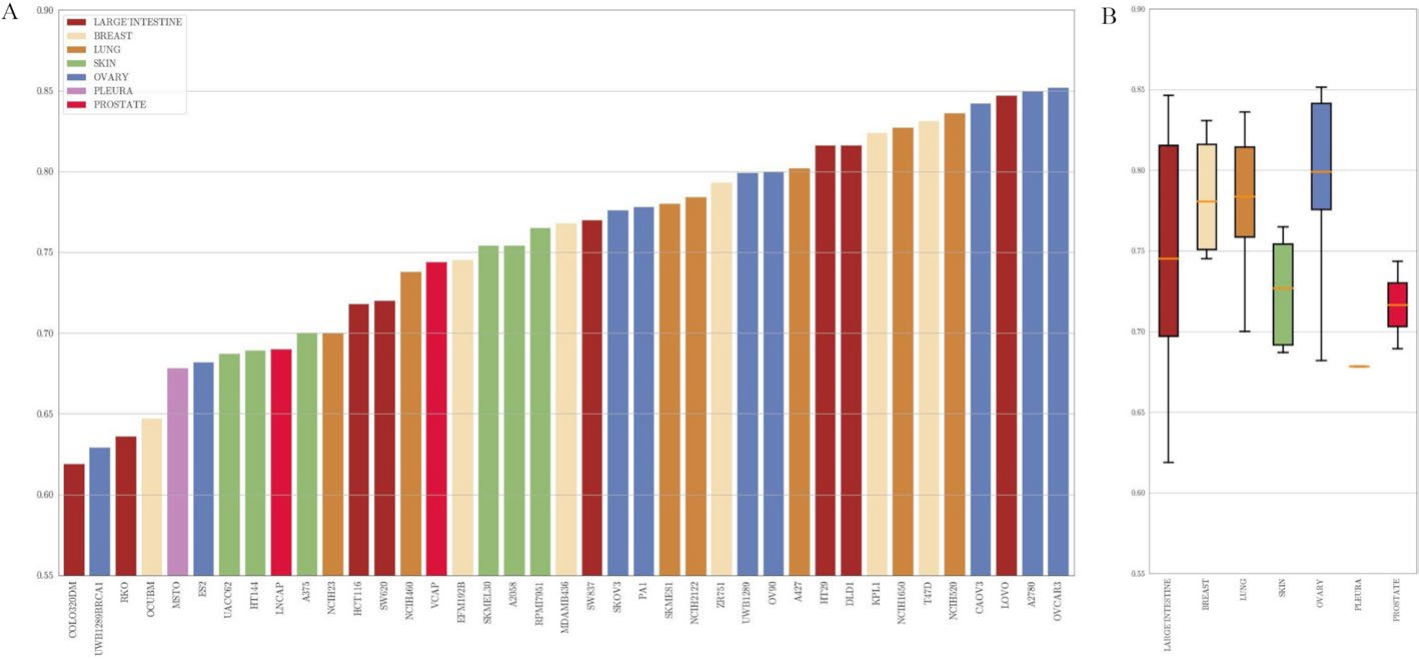
Performance across different tissues. **A** PCC values of each cell line. The tissue types of cell lines are represented by different colors. **B** The boxplot of the PCC values of each tissue. The orange horizontal line in each box indicates the median

### Performance across different tissues

Moreover, we visualize the performance of MTLSynergy across various cell lines and tissues based on PCC values. Figure 4A displays the PCC between the predicted synergy scores and the ground truth on each cell line, and the colors of the bars indicate the tissue types. The PCC of MTLSynergy across cell lines ranges from 0.62 (COLO320DM) to 0.85 (OVCAR3). Among the 39 cell lines, only 4 cell lines present a PCC lower than 0.65, whereas 23 cell lines (58.97%) exhibit a PCC higher than 0.75. We can see that cell lines from the same tissue exhibit a notable diversity in their PCC values. For example, the large intestine cell line COLO320DM diaplays the lowest PCC, but the large intestine cell line LOVO exhibits a PCC of 0.847, closely approaching the highest observed PCC.

As depicted in Fig. 4B, we further generate a boxplot illustrating the distribution of PCC values categorized by the tissue types of cell lines. Notably, cell lines associated with ovary tissue exhibit the highest median PCC value, while all tissues, with the exception of pleura, achieve median PCC values surpassing 0.70. Overall, synergy scores predicted by MTLSynergy show a strong correlation with the ground truth across various tissues, and no discernible association between PCC and tissue types is observed. Additionally, we also visualize the performance of MTLSynergy on each drug in Additional file 3.

**Table 3:** Results of Drug Synergy Prediction in the Ablation Study

Method	MSE	RMSE	PCC
MTLSynergy	**216.47±37.32**	**14.66±1.26**	**0.76±0.02**
OnlySynergy	235.24±39.20	15.29±1.25	0.74±0.02
MTLSynergy-NoAE	218.83±36.21	14.74±1.21	0.76±0.02
MTLSynergy-Regression	218.91±35.77	14.75±1.20	0.76±0.03

Boldface indicates the best value of each column in a particular metric

**Table 4:** Results of Monotherapy Sensitivity Prediction in the Ablation Study

Method	MSE	RMSE	PCC
MTLSynergy	**265.73±86.80**	**16.08±2.66**	0.66±0.08
OnlySensitivity	445.51±169.64	20.73±3.95	0.29±0.16
MTLSynergy-NoAE	272.31±80.34	16.32±2.45	**0.66±0.06**
MTLSynergy-Regression	280.19±72.81	16.60±2.18	0.63±0.06

Boldface indicates the best value of each column in a particular metric

### Ablation study

To elucidate the influence of multi-task learning and autoencoder on both the drug synergy prediction task and monotherapy sensitivity prediction task, we compare the prediction performance of MTLSynergy with several variants, including OnlySynergy, OnlySensitivity, and MTLSynergy-NoAE. Additionally, we introduce MTLSynergy-Regression that focuses solely on regression tasks to explore the effect of simultaneously performing regression and classification in a multi-task framework.

- **OnlySynergy**: This variant only performs the drug synergy prediction task. It combines the structure of the shared module and the drug combination synergy specific branch in MTLSynergy. And it employs the same output dimensions of the drug encoder and the cell line encoder as MTLSynergy.
- **OnlySensitivity**: This variant only performs the monotherapy sensitivity prediction task. It combines the shared module and the monotherapy sensitivity specific branch in MTLSynergy, and the output dimensions of the drug encoder and cell line encoder employed are consistent with MTLSynergy.
- **MTLSynergy-NoAE**: Compared with MTLSynergy, this variant removes the drug encoder and the cell line encoder. It directly inputs the drug features and cell line features into the predictor.
- **MTLSynergy-Regression**: It only performs the regression tasks of the drug synergy prediction and the monotherapy sensitivity prediction, with the same drug encoder and cell line encoder as MTLSynergy.

**Fig. 5:**
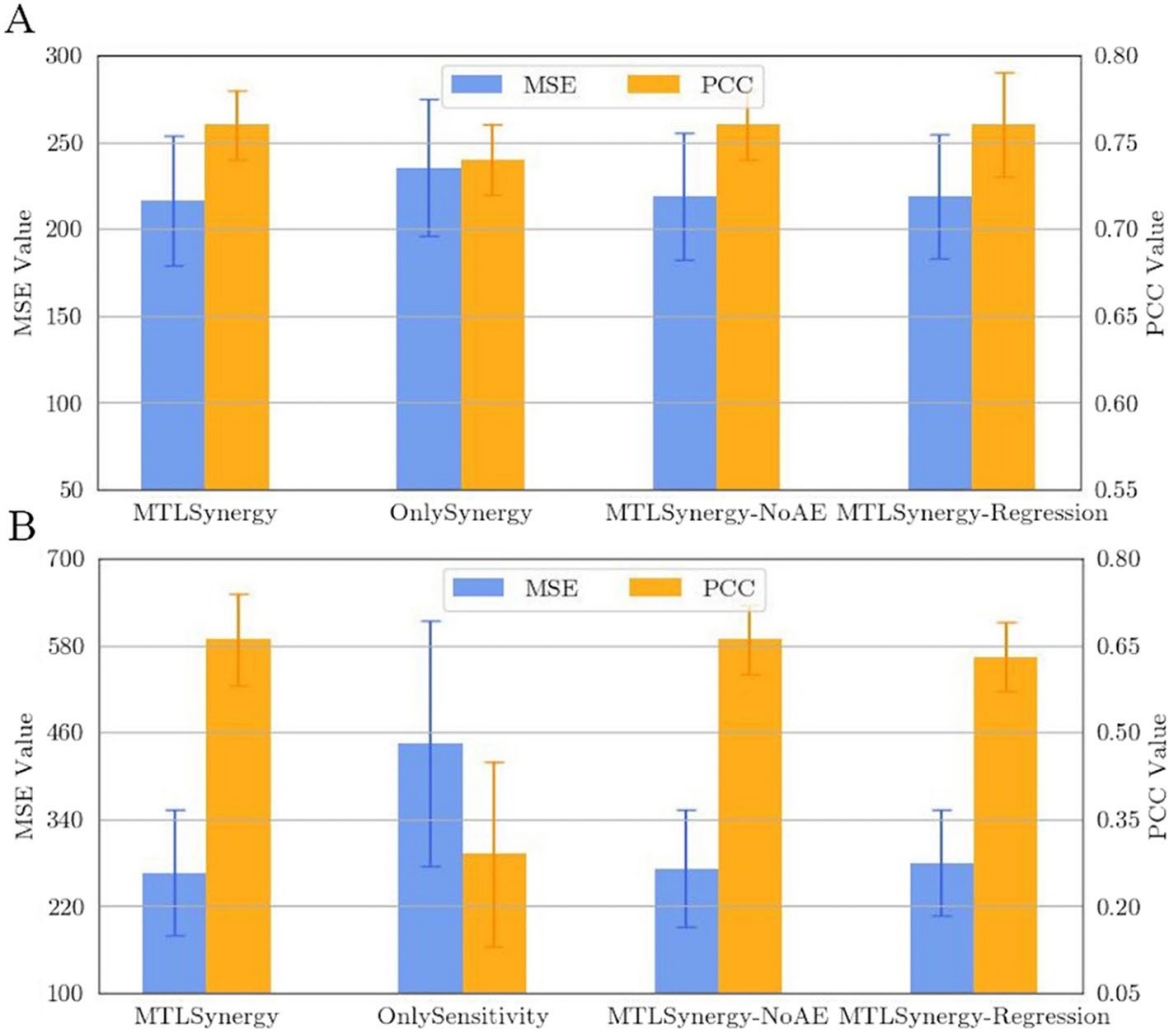
**A** Comparison of MTLSynergy and variants on the drug synergy prediction task. **B** Comparison of MTLSynergy and variants on the monotherapy sensitivity prediction task

Tables 3 and 4 present the results of the ablation study for drug synergy prediction task and monotherapy sensitivity prediction task. Compared with OnlySynergy and MTLSynergy-NoAE, MTLSynergy achieves the smallest MSE on the drug synergy prediction task. This observation implies that the inclusion of the monotherapy sensitivity prediction task has a positive influence on learning the response regularity of drug combinations on cell lines. In the multi-task joint learning process, the synergy prediction task effectively exploits the valuable insights from the sensitivity prediction task, resulting in the improvement of prediction performance. Figure 5A shows that MTLSynergy is outstandingly superior to OnlySynergy in terms of both MSE and PCC. Although the enhancement over MTLSynergy-NoAE may appear marginal, the integration of autoencoders to reduce the dimensionality of high-dimensional features holds significance. It captures essential feature information while substantially reducing training complexity.

On the monotherapy sensitivity prediction task, MTLSynergy exhibits a substantially smaller MSE compared to OnlySensitivity. It indicates the reciprocal influence of the sensitivity prediction and the synergy prediction on each other during training, underscored by the positive effect of multi-task learning on both tasks. Furthermore, when compared to MTLSynergy-NoAE, which lacks autoencoders, MTLSynergy achieves a reduced MSE and the same PCC. It signifies that the encoded representations generated through encoders are also beneficial for the sensitivity prediction task to some degree. Figure 5B visually reveals the advantages of MTLSynergy over other variants on the monotherapy sensitivity prediction task.

In terms of MSE and PCC, MTLSynergy-Regression exhibits a slight performance decline compared to MTLSynergy on the drug synergy prediction task and performs less favorably than MTLSynergy on the monotherapy sensitivity prediction task. However, the classification results obtained by applying a threshold to the predicted synergy scores from MTLSynergy-Regression, with a ROC AUC of 0.91 and PR AUC of 0.65, surpassing the classification results obtained directly from MTLSynergy. It’s noteworthy that many prior studies (like DeepSynergy, AuDnnSynergy, PRODeepSyn, etc.) employed thresholding based on regression scores to derive classification results. In these cases, the classification performance is strongly contingent on the quality of regression, thus placing a greater emphasis on enhancing regression performance. In our study, we attempt to incorporate the classification task into the multi-task network, aiming to achieve superior regression outcomes while concurrently delivering comparable and competitive classification results. Detailed results of variants on each fold are provided in Additional file 3.

**Fig. 6:**
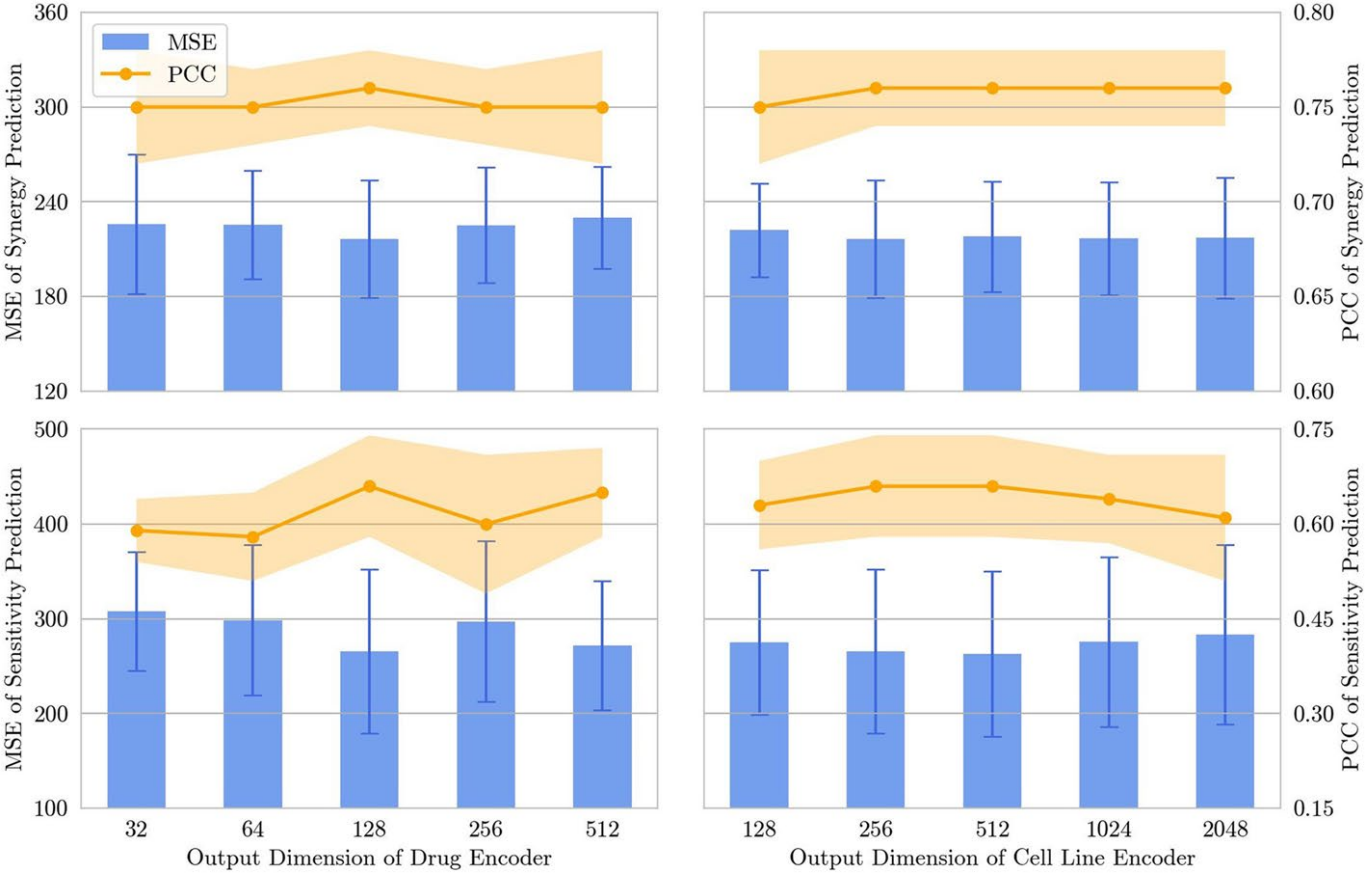
Results of hyperparameters sensitivity analysis

### Hyperparameters sensitivity analysis

To explore the effect of output dimensions of the drug encoder and the cell line encoder on the prediction performance of MTLSynergy, we conduct a hyperparameters sensitivity analysis experiment. In this analysis, we select the output dimension of the drug encoder $c_{drug}$ from {32, 64, 128, 256, 512}, and the output dimension of the cell line encoder $c_{cell}$ from {128, 256, 512, 1024, 2048}. We only modify one of $c_{drug}$ or $c_{cell}$ in each experiment, keep the other unchanged, and finally draw figures of MSE and PCC on the drug synergy prediction task and the monotherapy sensitivity prediction task concerning changes of $c_{drug}$ and $c_{cell}$ (Fig. 6).

We find that both prediction tasks in MTLSynergy are influenced by the output dimensions of the drug encoder and the cell line encoder, with the monotherapy sensitivity prediction task being notably sensitive to dimensionality changes. The first figure in the first row of Fig. 6 shows that MTLSynergy achieves the best MSE and PCC on the drug synergy prediction task when the output dimension of the drug encoder is set to 128. The second figure shows the fluctuations of MSE and PCC concerning the dimensionality changes of the cell line encoder. Notably, the prediction performance remains relatively stable when the dimension exceeds 128. For the monotherapy sensitivity prediction task, the first figure in the second row of Fig. 6 demonstrates substantial fluctuations on MSE and PCC when the dimension of the drug encoder changes. The optimal performance is achieved with a 128-dimensional drug encoder. The second figure indicates that MTLSynergy achieves the best result when the output dimension of the cell line encoder is set to 512, closely followed by the result with a 256-dimensional cell line encoder. We comprehensively consider the performance on two prediction tasks and the computational resources required for model training, and finally select a drug encoder and a cell line encoder with output dimensions of 128 and 256, respectively. Detailed results are provided in Additional file 3.

### Case study

We collect samples outside of the O’Neil dataset in DrugComb and find that the results of many cases predicted by MTLSynergy are consistent with previous *in vivo* and *in vitro* studies. For example, Amirouchene-Angelozzi et al. [33] reported that the drug combination of BEZ235 and RAD001 demonstrated the highest mean synergy score on melanoma cell lines in their combination screen experiments. MTLSynergy predicts a synergy score of 75.90 for this drug combination on the melanoma cell line A2058, signifying a high degree of synergy, consistent with their observation. In another study by Friedman et al. [34], it was noted that Vincristine and Erlotinib exhibited substantial synergy on multiple melanoma cell lines. The synergy scores computed by MTLSynergy for this combination on melanoma cell lines, including A2058, G-361, MEWO, SKMEL30, and SKMEL2, are 74.44, 47.62, 46.61, 38.36, and 36.57, respectively, all indicating strong synergy. These results align with their findings. Experiments conducted by Ma et al. [35] showed that the combination of Fulvestrant and MK2206 significantly increased apoptosis in the breast cancer cell line MCF7, while resistance developed when each drug was used in isolation. MTLSynergy computes a synergy score of 35.49, indicating the effectiveness of this combination for MCF7, which corresponds with their experimental results. Furthermore, Feliu et al. [36] conducted a clinical trial involving docetaxel combined with mitomycin C in patients with advanced non-small cell lung cancer (NSCLC). Their findings showed that the regimen had acceptable toxicity but did not achieve the intended improvement in efficacy. MTLSynergy assigns synergy scores of 0.07 and $-$2.45 for this drug combination on NSCLC cell lines NCI-H460 and EKVX, respectively, indicating additive and antagonistic effects, which corroborate the conclusions of their research. These illustrative cases underscore the practical utility of MTLSynergy in predicting novel synergistic drug combinations.

## Discussion and conclusion

In this paper, we propose a multi-task learning model named MTLSynergy, designed to simultaneously predict drug combination synergy and monotherapy sensitivity. We combine both regression and classification tasks for synergy and sensitivity predictions, thus obtaining regression scores and classification results within a single model. We also leverage autoencoders to perform feature dimensionality reduction, extracting essential information and enhancing learning efficiency. Our method begins with the pre-training of corresponding autoencoders utilizing drug and cell line features. Subsequently, we feed the encoded representations of drugs and cell lines obtained from the trained encoders into the predictor. This predictor comprises two key components: the shared module and the task-specific module. The shared module independently generates two representations for each drug–cell line pair. For the drug synergy prediction task, these two representations are concatenated and then forwarded to the drug combination synergy specific branch of the task-specific module. This branch ultimately produces the synergy score and the synergy probability distribution for a given drug combination. On the other hand, for the monotherapy sensitivity prediction task, only the first representation is utilized, feeding it into the monotherapy sensitivity specific branch of the task-specific module. The final outputs from this branch include the sensitivity score and the sensitivity probability distribution for a single drug.

Our experiments conducted on the large public dataset clearly demonstrate that MTLSynergy outperforms other SOTA methods in predicting drug synergy. Specifically, MTLSynergy exhibits significant advantages on the regression task while maintaining performance levels equivalent to those of the compared methods on the classification task. In addition, our ablation study provides compelling evidence that multi-task learning plays a pivotal role in enhancing both drug synergy prediction and monotherapy sensitivity prediction. When compared to single-task learning, multi-task learning proves to be highly effective in leveraging potentially valuable information from multiple related tasks, leading to superior learning outcomes. Furthermore, the inclusion of autoencoders in our method yields positive effects on prediction performance for both tasks compared with removing them. However, we need to carefully consider when determining the output dimensions of encoders. Our experiments exploring hyperparameters sensitivity reveal that MTLSynergy is sensitive to the dimensionality changes of encoders, thereby offering valuable guidance for model optimization. In our case study, we find that MTLSynergy’s predictions align with the outcomes of previous studies in many instances, which reflects the practical value of MTLSynergy. Overall, MTLSynergy represents a significant advancement in predicting anticancer synergistic drug combinations.

However, there exist some limitations of MTLSynergy. Firstly, our current method computes the total loss by directly summing the losses of multiple tasks. This may not represent the optimal solution for calculating the total loss in a multi-task model. How to find optimal weight parameters for each loss function, and how to combine multiple loss functions to achieve the best overall effect, are issues worth exploring when building a multi-task model. Secondly, we acknowledge that the performance of the monotherapy sensitivity prediction task in MTLSynergy has not been directly compared with previous studies. This lack of direct comparison arises from differences in datasets, measurements of sensitivity scores, and evaluation metrics adopted across studies. We expect to address these differences in future research to facilitate more meaningful comparisons in the sensitivity prediction task.

In summary, our study suggests that multi-task learning is beneficial for drug synergy prediction as well as monotherapy sensitivity prediction when integrating these two tasks into a single model. Compared with SOTA methods, the ability of MTLSynergy to discover new anticancer synergistic drug combinations is notably improved by incorporating other relative tasks, which shows a certain reference value for the follow-up research. MTLSynergy is expected to be a useful tool to pre-screen anticancer synergistic drug combinations.

### Supplementary Information

**Additional file 1**. Detail information of 3118 drugs.

**Additional file 2**. Detail information of 175 cell lines.

**Additional file 3**. More experimental data.

## Data Availability

The dataset supporting the conclusions of this article is available in the MTLSynergy repository, https://github.com/TOJSSE-iData/MTLSynergy/tree/main/data. The source code is available at https://github.com/TOJSSE-iData/MTLSynergy.

## Abbreviations

HTS: High-throughput screening
ML: Machine learning
DL: Deep learning
MTL: Multi-task learning
DNNs: Deep neural networks
GCNs: Graph convolutional neural networks
FC: Fully connected
SOTA: State-of-the-art
MSE: Mean squared error
RMSE: Root mean squared error
PCC: Pearson correlation coefficient
ROC-AUC: Area under the receiver operating characteristic curve
PR-AUC: Area under the precision–recall curve
ACC: Accuracy
