# Supplementary material for: Predicting anticancer synergistic drug combinations based on multi-task learning
Source: BMC Bioinformatics. 2023 Nov 27;24:448. doi: 10.1186/s12859-023-05524-5 (PMC10680313; doi:10.1186/s12859-023-05524-5)
Supplement: Supplementary file 3 — Additional file 3. More experimental data. [file 12859_2023_5524_MOESM3_ESM.pdf]

Predicting Anticancer Synergistic Drug Combinations Based on Multi-task Learning

Supplementary Information

Danyi Chen[1], Xiaowen Wang[1], Hongming Zhu[1], Yizhi Jiang[1], Yulong Li[1], Qi Liu[2] and Qin Liu[1]

[1] School of Software Engineering, Tongji University, Shanghai, 201804, China

[2] Key Laboratory of Spine and Spinal Cord Injury Repair and Regeneration (Tongji University), Ministry of Education, Orthopaedic Department of Tongji Hospital, Bioinformatics Department, School of Life Sciences and Technology, Tongji University, Shanghai, 200092, China.

This report gives supplementary information to the manuscript "Predicting Anticancer Synergistic Drug Combinations Based on Multi-task Learning". It provides more detailed experimental data.

We adopt 5-fold nested cross-validation in the experiments, the process is as follows:

In the 5-fold nested cross-validation, we employ 4-folds (3-folds for inner training and the other for inner validating) to select the optimal hyperparameters in the inner loop based on the validation loss. In the outer loop, we train the model with searched optimal hyperparameters on these 4-folds, and the 4-folds are randomly split into an outer training set and an outer validation set in a ratio of 9:1. The remaining fold is utilized as a test set to evaluate the trained model. In this process, every fold is selected as the test set in turn, so the results we report include the mean and standard deviation on the 5-fold data.

The hyperparameters of the two machine learning methods to be searched and their candidate values are listed in Table S1.

Table S1: Hyperparameters and candidate values for machine learning models

Methods	Hyperparameter	Candidate Values
Gradient Tree Boost	number of estimators	{128, 512, 1024}
Gradient Tree Boost	learning rate	{0.1, 0.05, 0.01}
Random Forest	number of estimators	{128, 512, 1024}
Random Forest	number of features (n)	$\{\sqrt{n}, 256, 512\}$

Tables S2-S11 show the results of different methods on different folds.

Table S2: Synergy prediction results of MTLSynergy on each fold.

	MSE	RMSE	PCC	ROC-AUC	PR-AUC	ACC
Fold 0	175.4085	13.2442	0.7663	0.9218	0.5765	0.9542
Fold 1	195.9944	13.9998	0.7529	0.8870	0.6146	0.9421
Fold 2	254.9323	15.9666	0.7364	0.8985	0.6505	0.9310
Fold 3	267.4725	16.3546	0.7568	0.8652	0.5769	0.9288
Fold 4	188.5539	13.7315	0.8054	0.9080	0.6954	0.9328
Mean	216.47±37.32	14.66±1.26	0.76±0.02	0.90±0.02	0.62±0.05	0.94±0.01

Table S3: Synergy prediction results of Gradient Tree Boosting on each fold.

	MSE	RMSE	PCC	ROC-AUC	PR-AUC	ACC
Fold 0	225.0030	15.0001	0.6880	0.9125	0.5254	0.9525
Fold 1	244.3021	15.6302	0.6768	0.8973	0.5287	0.9343
Fold 2	292.7060	18.7923	0.6593	0.8904	0.5731	0.9164
Fold 3	353.1500	18.7923	0.6593	0.8767	0.5198	0.9151
Fold 4	259.2199	16.1003	0.7200	0.9112	0.6407	0.9226
Mean	274.88±44.97	16.53±1.33	0.69±0.02	0.90±0.01	0.56±0.05	0.93±0.01

Table S4: Synergy prediction results of Random Forest on each fold.

	MSE	RMSE	PCC	ROC-AUC	PR-AUC	ACC
Fold 0	308.5514	17.5656	0.5328	0.8924	0.5342	0.9381
Fold 1	306.1467	17.4970	0.5733	0.8557	0.4515	0.9105
Fold 2	382.7360	19.5636	0.5684	0.8732	0.4844	0.9083
Fold 3	444.2717	21.0778	0.5591	0.8527	0.5108	0.9034
Fold 4	359.0564	18.9488	0.5953	0.8882	0.6024	0.9076
Mean	360.15±51.32	18.93±1.34	0.57±0.02	0.87±0.02	0.52±0.05	0.91±0.01

Table S5: Synergy prediction results of OnlySynergy on each fold.

	MSE	RMSE	PCC	ROC-AUC	PR-AUC	ACC
Fold 0	206.4844	14.3696	0.7179	0.9191	0.5628	0.9538
Fold 1	204.1599	14.2885	0.7402	0.8966	0.5567	0.9357
Fold 2	259.6594	16.1140	0.7252	0.9069	0.6418	0.9288
Fold 3	301.2561	17.3567	0.7188	0.8671	0.5415	0.9233
Fold 4	204.6378	14.3052	0.7845	0.9092	0.6611	0.9278
Mean	235.24±39.20	15.29±1.25	0.74±0.02	0.90±0.02	0.59±0.05	0.93±0.01

Table S6: Synergy prediction results of MTLSynergy-NoAE on each fold.

	MSE	RMSE	PCC	ROC-AUC	PR-AUC	ACC
Fold 0	181.1702	13.4599	0.7579	0.9154	0.5757	0.9541
Fold 1	209.1605	14.4624	0.7430	0.8746	0.5614	0.9390
Fold 2	245.3237	15.6628	0.7426	0.9109	0.6652	0.9324
Fold 3	274.6712	16.5732	0.7463	0.8682	0.5891	0.9296
Fold 4	183.8437	13.5589	0.8077	0.8981	0.6832	0.9318
Mean	218.83±36.21	14.74±1.21	0.76±0.02	0.89±0.02	0.61±0.05	0.94±0.01

Table S7: Synergy prediction results of MTLSynergy-Regression on each fold.

	MSE	RMSE	PCC	ROC-AUC	PR-AUC	ACC
Fold 0	180.7203	13.4432	0.7600	0.9365	0.6320	0.9607
Fold 1	208.7060	14.4467	0.7419	0.9045	0.6229	0.9394
Fold 2	251.1539	15.8478	0.7379	0.9266	0.6856	0.9319
Fold 3	269.7996	16.4256	0.7537	0.8837	0.5932	0.9273
Fold 4	184.1693	13.5709	0.8096	0.9197	0.7036	0.9332
Mean	218.91±35.77	14.75±1.20	0.76±0.03	0.91±0.02	0.65±0.04	0.94±0.01

Table S8: Sensitivity prediction results of MTLSynergy on each fold.

	MSE	RMSE	PCC	ROC-AUC	PR-AUC	ACC
Fold 0	176.0134	13.2670	0.7836	0.8220	0.2944	0.8934
Fold 1	399.2413	19.9810	0.6672	0.7640	0.4997	0.8085
Fold 2	170.0227	13.0393	0.5665	0.7944	0.2592	0.9427
Fold 3	317.0677	17.8064	0.6911	0.8203	0.5235	0.8348
Fold 4	266.3133	16.3191	0.5966	0.7050	0.3568	0.8104
Mean	265.73±86.80	16.08±2.66	0.66±0.08	0.78±0.04	0.39±0.11	0.86±0.05

Table S9: Sensitivity prediction results of OnlySensitivity on each fold.

	MSE	RMSE	PCC	ROC-AUC	PR-AUC	ACC
Fold 0	287.1779	16.9463	0.5414	0.6554	0.1604	0.8323
Fold 1	703.5831	26.5251	0.0347	0.5252	0.2175	0.7410
Fold 2	277.1706	16.6484	0.3170	0.7230	0.1767	0.9473
Fold 3	583.5730	24.1573	0.2281	0.6532	0.2394	0.7730
Fold 4	376.0544	19.3921	0.3461	0.6599	0.3524	0.8226
Mean	445.51±169.64	20.73±3.95	0.29±0.16	0.64±0.06	0.23±0.07	0.82±0.07

Table S10: Sensitivity prediction results of MTLSynergy-NoAE on each fold.

	MSE	RMSE	PCC	ROC-AUC	PR-AUC	ACC
Fold 0	203.6432	14.2704	0.7406	0.6968	0.1632	0.8603
Fold 1	388.7951	19.7179	0.6984	0.8519	0.7006	0.8387
Fold 2	170.0918	13.0419	0.5700	0.9113	0.3281	0.9467
Fold 3	331.5820	18.2094	0.6768	0.7513	0.4413	0.8142
Fold 4	267.4515	16.3539	0.6243	0.7220	0.3457	0.8214
Mean	272.31±80.34	16.32±2.45	0.66±0.06	0.79±0.08	0.40±0.18	0.86±0.05

Table S11: Sensitivity prediction results of MTLSynergy-Regression on each fold.

	MSE	RMSE	PCC	ROC-AUC	PR-AUC	ACC
Fold 0	233.0286	15.2653	0.6714	0.7879	0.3537	0.9082
Fold 1	389.0277	19.7238	0.6537	0.8430	0.6514	0.8034
Fold 2	181.7615	13.4819	0.5216	0.7780	0.2666	0.9517
Fold 3	330.7737	18.1872	0.7011	0.8800	0.5805	0.8256
Fold 4	266.3392	16.3199	0.6229	0.8084	0.4846	0.8471
Mean	280.19±72.81	16.60±2.18	0.63±0.06	0.82±0.04	0.47±0.14	0.87±0.05

We also list the detailed results of MTLSynergy on different dimensions. The output dimension of the drug encoder c_{drug} is selected from {32, 64, 128, 256, 512}, and the output dimension of the cell line encoder c_{cell} is chosen from {128, 256, 512, 1024, 2048}. We only modify one of c_{drug} or c_{cell} in each experiment and keep the other unchanged.

Table S12: Results of MTLSynergy on different dimensions.

Dimension Combinations	Synergy Prediction		Sensitivity Prediction	
	MSE	PCC	MSE	PCC
c_{drug}=32, c_{cell}=256	225.82±44.16	0.75±0.03	307.69±62.66	0.59±0.05
c_{drug}=64, c_{cell}=256	225.54±34.32	0.75±0.02	298.71±79.20	0.58±0.07
c_{drug}=128, c_{cell}=256	216.47±37.32	0.76±0.02	265.73±86.80	0.66±0.08
c_{drug}=256, c_{cell}=256	225.16±36.69	0.75±0.02	297.21±84.88	0.60±0.11
c_{drug}=512, c_{cell}=256	230.01±32.36	0.75±0.03	271.71±68.29	0.65±0.07
c_{drug}=128, c_{cell}=128	222.06±29.65	0.75±0.03	275.37±76.18	0.63±0.07
c_{drug}=128, c_{cell}=512	217.91±35.07	0.76±0.02	262.89±87.19	0.66±0.08
c_{drug}=128, c_{cell}=1024	216.62±35.82	0.76±0.02	275.59±89.75	0.64±0.07
c_{drug}=128, c_{cell}=2048	217.07±38.22	0.76±0.02	283.47±94.75	0.61±0.10

We evaluate MTLSynergy, DeepSynergy, Gradient Tree Boosting, and Random Forest using the same feature data on the *Leave Drugs Out* scenario (samples are split to make that drugs seen in the test set are not in the training set) and on the *Leave Cell Lines Out* scenario (samples are split to make that cell lines seen in the test set are not in the training set), respectively. The detailed results are shown in Tables S13 and S14.

Table S13: Results of the method comparison in the *Leave Drug Out* scenario

Method	MSE	RMSE	PCC
Gradient Tree Boosting	429.90±23.63	20.73±0.57	0.45±0.03
Random Forest	450.18±23.84	21.21±0.57	0.39±0.03
DeepSynergy	454.07±24.81	21.30±0.86	0.44±0.03
MTLSynergy	461.03±33.60	21.46±0.79	0.40±0.04

Table S14: Results of the method comparison in the *Leave Cell Line Out* scenario

Method	MSE	RMSE	PCC
Gradient Tree Boosting	378.93±154.34	19.07±3.92	0.54±0.08
Random Forest	388.39±141.55	19.39±3.54	0.51±0.06
MTLSynergy	399.04±155.49	19.60±3.84	0.51±0.08
DeepSynergy	509.91±123.07	22.40±2.89	0.47±0.07

Figure S1 summarizes the PCC between the predicted scores and the ground truth on each drug, and the colors of bars shows the targets of drugs. The PCC of MTLSynergy across drugs ranges from 0.58 (DINACICLIB) to 0.83 (ETOPOSIDE). Among the 38 drugs, only 4 drugs present a PCC lower than 0.65, whereas 18 drugs (47.37%) exhibit a PCC higher than 0.75. There is no clear association between targets and correlation can be observed. 0.70. Overall, synergy scores predicted by MTLSynergy show a strong correlation with the ground truth in different drugs, and no clear association between PCC and targets is observed.

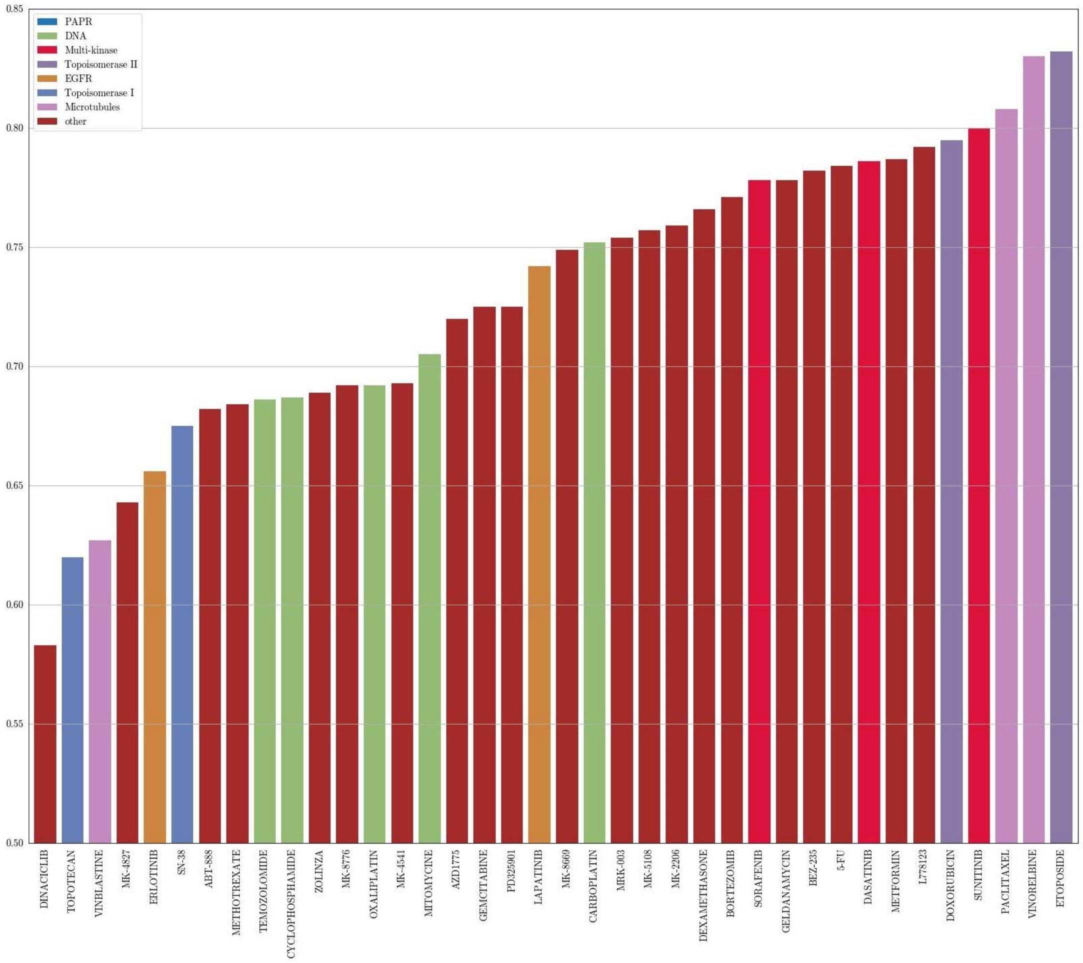

Figure S1: PCC values of each drug. The color of the bar represents the target of the drug.
